# Sexual and mental health disparities among young sexual minority women compared to exclusively heterosexual women: a national study

**DOI:** 10.3389/fgwh.2025.1591604

**Published:** 2026-01-20

**Authors:** Diana Fernandes, Lorraine Chok, Camille Béziane, Yara Barrense-Dias

**Affiliations:** 1 Department of Epidemiology and Health Systems, Center for Primary Care and Public Health (Unisanté), University of Lausanne, Lausanne, Switzerland; 2 Association Les Klamydia’s, Lausanne, Switzerland

**Keywords:** sexual and gender minorities, sexual health, sexual violence, sexual behavior, health education

## Abstract

**Introduction:**

Young sexual minority women (YSMW)'s sexual health is often overlooked in research, with most studies focusing on men who have sex with men or transgender women.

**Methods:**

This study compares the sexual and mental health of young lesbians and bisexual women with exclusively heterosexual women using data from a 2017 Swiss study on young adults’ sexual health and behaviors. The sample includes 2,316 sexually active cisgender women. Bivariate analyses were conducted, followed by a multinomial regression using exclusively heterosexual women as the reference group.

**Results:**

Sexual orientation is associated with STI diagnosis, HIV testing, age at first gynecological visit, protection at last intercourse, intercourse involving multiple partners and sexual violence. At the multivariate level, lesbians are more likely to use no protection, to be older at their first gynecological visit, to have experienced three-way intercourse and to smoke. Bisexual women are more likely to use no protection, to report STI diagnosis, to be victims of sexual abuse, and to have experienced intercourse involving multiple partners.

**Conclusion:**

Further research and inclusive sexual health education and prevention campaigns are urgently needed to provide inclusive, comprehensive information on topics such as same-gender relationships, bisexual behaviors to reduce disparities in sexual and mental health outcomes.

## 1 Introduction

As adolescents and young adults navigate their first sexual experiences, some do not receive accurate or comprehensive information regarding safer sex. For LGBTQIA+ youth, stigmatization, limited access to educational resources, and societal taboos surrounding sexual education may exacerbate this lack of information (1, 2). This challenge may also extend to young non-heterosexual women, referred to as young sexual minority women (YSMW) which specifically denotes women identifying as bisexual or lesbian. In this study, the terms woman or women refers to cisgender woman or women. These women may not always be aware of their sexual health risks (3), potentially increasing their vulnerability while exploring their sexuality. For example, women who have sex exclusively with women (WSW) report infrequent use of protective barriers during sexual encounters. A 2019 study conducted in French-speaking Switzerland found that only 13% of WSW consistently used protection during sexual intercourse with another woman or AFAB (assigned female at birth) partners, while 70.8% never used any type of protective barrier (4, 5). This could be explained by the wide misconception that WSW are not at risk of sexually transmitted infections (6). Another explanation put forth (7) in a scoping review on the gaps in sexual health research is the “heteronormative model of health care”. This model perpetuates inequalities in the care of SMW by not adequately taking into account their sexual practices.

In an Australian study conducted among 25–30-year-old women (8), women who have sex with both men and women reported higher rates of STI diagnoses compared to their exclusively heterosexual or homosexual counterparts, potentially due to a greater number of male sexual partners (9).

YSMW appear to engage in higher rates of risk behaviors, including sexual ones such as inconsistent use of protection, a greater number of casual sexual partners (10, 11), and more frequent substance use during sexual intercourse (12). Adolescents and young adults are more likely to consume psychoactive substances, such as alcohol, which may increase their risk for STI (13). In addition, substance use is often linked to mental health issues, and although LGBTQIA+ populations generally report having poorer mental health (8), bisexual women are the most disadvantaged compared to heterosexual or lesbian women (8, 10, 12). Indeed, as shown in a literature review (2018), compared to heterosexual women, SMW usually present higher rates of depression and anxiety. This has been explained by the minority stress theory which “expects mental health disparities for people with minority sexual orientations compared with heterosexuals” (14) due to the specific stigma faced by sexual minorities. For SMW, however, the discrimination experienced not only stems from their sexual minority status, as well as their status as women (15). Furthermore, compared to lesbians, bisexual women tend to present equal or higher rates of depression and anxiety which can be attributed to “sexual orientation-based discrimination, bisexual invisibility and erasure, and a lack of bisexual-affirmative support”. Furthermore, bisexual women report higher rates of sexual victimization compared to both heterosexual and lesbian women (16, 17). Finally, a lot of mental health research on sexual minorities has focused primarily on sexual minority men which could account for the lack of understanding and services adapted to the needs of SMW (18).

YSMW may also feel excluded from STI prevention campaigns, especially if they do not, or rarely, engage in sexual encounters with men (6, 19, 20). Prevention efforts have historically focused on men who have sex with men (MSM) and HIV. Similarly, discussions with health professionals often prioritize pregnancy prevention while underemphasizing STI prevention, which is predominantly framed around the use of external condoms (2, 21).

Despite these disparities, the sexual health of YSMW remains understudied. Most research (9, 22, 23) focuses on MSM and transgender women, while cisgender bisexual and lesbian women are underrepresented (11). The aim of this study is to describe the characteristics of young YSMW in Switzerland to provide a comprehensive overview of their sexual health, using heterosexual women as a comparison group.

## 2 Methods

### 2.1 Participants

Data for this study were drawn from the 2017 Swiss national study on sexual health and behaviors among young adults (24). A representative sample of 24–26-year-olds (as of September 30, 2016) was randomly selected by the Swiss Federal Statistical Office. An invitation letter was sent out to each participant with a link that led them to the questionnaire through which the data was collected. This survey addressed various facets of youth's sexual health and behaviors (24). This age range was chosen to ensure that most participants would already be sexually active while still being young enough to recall their first sexual experiences accurately. At the time of the survey, the mean participant age was 26.3 years, as data collection occurred between June and November 2017. Out of the 7,142 participants (response rate 15.1%), 5,181 completed the entire questionnaire. For this present study, we included only cisgender women who were sexually active, excluding those who reported never having engaged in any form of sexual intercourse with a partner. The final analytical sample consisted of 2,316 sexually active women.

### 2.2 Measures

#### 2.2.1 Dependent variable

Respondents were asked how they self-identified their sexual orientation (25). Responses included: heterosexual, lesbian or gay, bisexual, I don't know or I'm not sure, I don't want to answer, other. For this study, the variable was recoded into three categories: heterosexual, lesbian, and bisexual. Respondents who selected I don't know, I'm not sure, I don't want to answer were classified as missing values. All women in the sample were cisgender.

#### 2.2.2 Independent variable

##### 2.2.2.1 Sociodemographic variables

Sociodemographic characteristics included birthplace (Switzerland/other), education level (apprenticeship/secondary/higher/other), family socio-economic status (SES) as perceived at age 15 (above average/average/below average), inspired by the ESPAD study (26), and age at the time of the survey.

##### 2.2.2.2 Sexual health

Respondents were asked whether they had ever been diagnosed with a STI by a healthcare professional and could answer yes/no/I don't know/I don't want to answer. Responses of “I don't know” and “I don't want to answer” (*n* = 135) were treated as missing values. Participants who answered yes were asked to specify the STI from a list including gonorrhea, chlamydia, syphilis, HPV, herpes, or other (free text). HIV testing was inquired independently (24), with responses similarly grouped (*n* = 1,769). HPV vaccination status was recorded with the same response options (*n* = 1,770). Participant also reported whether they had ever consulted a gynecologist (yes/no) and their age at their first visit.

Participants were asked whether they had sought information on sexual issues (yes/no) and whether the information was useful (a lot/a little/not at all/I don't know). They also identify their primary source of sex education during adolescence from options including parents (combined responses for mother and father), school, friends, no one, internet, other (free text).

##### 2.2.2.3 Sexual behaviors

Regarding sexual behaviors, participants reported the age at which they first engaged in sexual (with or without vaginal/anal penetration, oral sex, etc.). A series of questions were then selected to measure sexual behaviors presenting an elevated STI risk such as the contraception/protection used during the last intercourse, the lifetime number of sexual partners, and participation in group sexual activities such as three-way or intercourse involving more than three people (27).

Contraception/protection use during the last intercourse was categorized from multiple-choice options: nothing, external condom, internal condom, contraceptive pill, hormonal vaginal ring, contraceptive patch, hormonal implant or injection, hormonal or copper intrauterine device, spermicides, withdrawal, natural methods (body temperature, ovulation prediction), other (free text option), I don't remember. Participants selecting “I don't remember” were excluded from analysis. Responses were grouped into three categories: “Condom ± Contraceptive” included barrier and protective methods (external and internal condom) with or without the use of a contraception), “Contraceptive” contained only contraception without condom, and “Non-Use” grouped low effective contraceptive methods (such as withdrawal and natural methods) and non-use (27). Multivariate analyses were controlled for the nature of the current relationship, as a possible explanation for the non-use of protection at last intercourse could be a steady relationship.

The lifetime number of sexual partners was assessed in two separate questions: one regarding total sexual partners without distinction, and another specifically about casual sexual partners. Responses were grouped into the following categories: 1–3, 4–7, 8 or more (28). Participation in group sexual activities was measured as lifetime experience of sexual intercourse involving three or more partners and dichotomized into yes (often/sometimes/rarely) or no (never).

##### 2.2.2.4 Sexual violence

Sexual violence was measured using three questions. Sexual compliance: responded yes to the question Have you ever accepted sexual intercourse without really wanting? (*N* = 1,312, 24.8%); Unwanted Sexual Intercourse or Contacts: responded “yes” to the question During your lifetime, were some of your sexual contacts or intercourse unwanted? (*N* = 505, 9.6%); and Sexual assault: responded yes to the question Have you ever been sexually assaulted or abused? (*N* = 489, 9.2%) (29).

##### 2.2.2.5 Substance use and mental health

Substance use was assessed for the past 30 days, including alcohol, cigarettes, cannabis, and other illegal drugs, with responses dichotomized into yes or no (24). Participants also reported whether they had engaged in sexual activity while under the influence of drugs or alcohol. Mental health was assessed using the Mental Health Inventory (MHI-5) over the last 4 weeks (24). The scale consists of 5 items, scored from 0 to 100, with higher scores indicating better mental health. Following the Swiss health observatory standards, a score lower or equal to 52 was used to identify participants with poor mental health (30, 31).

#### 2.2.3 Data analysis

First, we conducted bivariate analyses to compare the three groups (heterosexual, bisexual, and lesbian women). Chi-square tests were used for categorical variables and Student's t-tests were employed for continuous variables. Second, a multinomial regression analysis was performed on variables that were statistically significant at the bivariate level, using heterosexual women as the reference. Results are presented as adjusted odds ratios (aOR) with 99% confidence intervals (CI), given the smaller sample sizes of bisexual (*n* = 106) and lesbian (*n* = 30) woman. Lowering the significance level to 1% reduces the risk of false positives (Type I errors), which is particularly important when comparing small samples to a larger one, as it ensures more robust conclusions despite the higher variability in smaller datasets. However, we reserve the right to comment on results with a significance level of less than 5% as trends, acknowledging their potential relevance while maintaining a cautious interpretation.

We also ran additional analyses combining bisexual and lesbian women into a single category for increased statistical power and because they may share common experiences as YSMW.

Since females from the French-speaking part of Switzerland were overrepresented in the initial study, data were weighted for two characteristics to ensure the most representative sample possible: canton (or region) of residence and sex assigned at birth. These two characteristics were selected because their distributions are officially recorded and available through national household statistics and surveys conducted by the Swiss Federal Statistical Office.

All analyses were conducted using STATA 17.0 (StataCorp, College Station, TX, USA).

## 3 Results

### 3.1 Bivariate analyses

#### 3.1.1 Sociodemographic variables

In the final sample of 2,316 cisgender women, 103 (4.4%) identified as bisexual, 28 (1.2%) identified as lesbian and 2,185 (94.3%) identified as heterosexual. No significant differences were found among the groups for birthplace, socioeconomical status, education level and age.

#### 3.1.2 Sexual health

Significant differences were observed in STI diagnoses (Table 1). A quarter of bisexual women (26.4%) reported having been diagnosed with an STI, compared to 13,4% of heterosexual women and 5.8% of lesbians. When examining specific STIs, a trend was observed for chlamydia (*p* < .05) with 28.9% of bisexual women reporting a diagnosis compared to 16.9% of heterosexual women and 0.0% of lesbians. Bisexual women were more likely to report having been tested for HIV (62.2%), while lesbians were the least likely to report having been tested (38.5%). A difference was observed regarding whether individuals had ever consulted a gynecologist in their lifetime, with lesbians reporting the lowest rates, while heterosexual and bisexual women had similar rates close to 100%. In addition to this difference, the age at which the first visit occurred was significantly later for lesbians compared to the other groups, representing an approximate three-year delay.

**Table 1:** Results of the bivariate analyses.

	Bivariate analyses comparing the three groups				Bivariate analyses comparing heterosexual women to YSMW	
	Heterosexual women (%) *N* = 2,185	Lesbians (%) *N* = 28	Bisexual women (%) *N* = 103	*p*-value	YSMW (%) *N* = 131	*p*-value
Sociodemographic variables						
Age (mean ± s.e.)	26.3 ± .02	26.4 ± .16	26.4 ± .08	ns	26.4 ± .07	ns
Birthplace (Switzerland)	88.4	87.4	82.3	ns	83.4	ns
Family SES at age 15				ns		ns
Above average	19.6	11.9	20.3		18.6	
Average	64.5	68.8	56.7		59.2	
Below average	16.0	19.3	23.0		22.2	
Education level				ns		<.05
Apprenticeship	19.4	28.9	26.0		26.6	
Secondary	19.9	24.8	24.4		24.5	
Higher	56.3	40.5	43.9		43.1	
Other	4.3	5.8	5.7		5.7	
Sexual health						
Ever received STI diagnosis	13.4	5.8	26.4	<.01	21.9	<.01
Chlamydia^a^	16.9	0.0	28.9	<.05	25.5	Ns
Gonorrhea^a^	0.9	11.0	0.0	<.01	1.3	Ns
Syphilis^a^	0.1	0.0	0.0	Ns	0.0	Ns
HPV^a^	15.0	0.0	20.1	Ns	17.8	Ns
Herpes^a^	9.8	0.0	9.4	Ns	8.4	Ns
Other^a^	7.2	16.6	7.0	ns	8.1	Ns
Ever tested for HIV	48.6	38.5	62.2	<.01	56.9	Ns
HPV vaccine	51.5	42.4	51.8	ns	50.1	Ns
Gynaecologists visit at least once	97.4	86.6	96.5	<.01	94.4	<.05
Age at first gynaecologist visit (mean ± s.e.)	16.8 ± .06	19.4 ± .55	16.3 ± .28	<.01	16.9 ± .26	Ns
Sexual behaviors						
Age at first sexual experience (mean ± s.e.)	16.5 ± .06	17.1 ± .47	15.9 ± .27	ns	16.1 ± .24	ns
Contraception/protection last intercourse				<.01		<.01
Condom ± Contraceptive	51.2	9.3	37.5		31.5	
Contraceptive	39.9	3.2	36.8		29.7	
None	8.9	87.5	25.7		38.8	
Number of lifetime sex. partners				<.01		<.01
1–3	41.1	34.1	17.9		21.4	
4–7	26.5	24.8	25.5		27.5	
8 or more	32.4	31.0	56.6		51.1	
Number of occasional sex. partners				<.01		<.01
0–3	68.8	73.1	48.4		53.7	
4–7	16.3	9.1	24.8		21.4	
8 or more	14.9	17.8	26.8		24.8	
Three-way	7.6	19.6	33.7	<.01	30.7	<.01
More than 3 sexual partners at once	1.7	0.0	9.8	<.01	7.7	<.01
Sexual intercourse under the influence of drugs or alcohol	45.1	41.4	58.1	<.05	54.5	<.05
Looked for sexual information	23.2	15.3	32.3	<.05	28.7	
The information was useful (*n* = 545 had ever looked for sexual information)				ns		ns
A lot	31.0	22.9	32.4		31.3	
A little	57.7	77.1	54.8		57.4	
Not at all	9.1	0.0	10.2		9.0	
I don't know	2.1	0.0	2.6		2.3	
Main source of sex education during adolescence				<.05		ns
Parents	31.3	31.9	29.1		29.7	
School	16.4	14.9	14.1		14.3	
Friends	37.0	26.4	39.2		36.5	
No one	2.4	0.0	6.7		5.2	
Internet	5.3	18.4	5.0		7.9	
Other	7.5	8.4	5.8		6.4	
Sexual violence						
Sexual abuse	14.5	16.2	39.7	<.01	34.6	<.01
Regretted intercourse	51.4	48.1	74.5	<.01	68.8	<.01
Intercourse without wanting to	54.5	34.9	64.9	<.01	58.4	ns
Substance use and mental health						
Current cigarettes smoker (yes)	38.6	68.8	64.6	<.01	65.5	<.01
Alcohol misuse 30 days (yes)	26.4	25.3	30.3	ns	29.2	ns
Cannabis use 30 days (yes)	9.2	15.0	20.7	<.01	19.5	<.01
Illegal drugs 30 days (yes)	2.0	6.5	4.1	ns	4.6	<.05
Mental health (poor)	16.7	26.5	24.6	<.05	25.0	<.01

ns, non-significant; s.e., standard errors; YSMW, young sexual minority women (grouping lesbians and bisexual women).

a The percentages presented for each STI refer to individuals who reported having previously received a diagnosis: 285 heterosexual, 2 bisexual, and 26 bisexual or 28 YSMW.

#### 3.1.3 Sexual behaviors

Lesbians were significantly more likely to report not using any form of protection or contraceptives, with 87.5% indicating no use, compared to 25.7% of bisexual women and 8.9% of heterosexual women. Significant differences were also observed in the number of sexual partners across groups, with bisexual women reporting the highest rates for both overall and casual partners. YSMW reported higher rates of engaging in three-way sexual encounters compared to heterosexual women, with bisexual women being the most likely to report such experiences, followed by lesbians. Sexual encounters involving more than three partners were also more commonly reported among bisexual women, while such encounters were rare among heterosexual women and not reported by lesbians.

Trends were observed (*p* < .05) for sexual intercourse under the influence of substances, seeking information regarding sexual issues and lack of sex education resources, with bisexual women reporting higher rates. No significant differences were found for the age at first sexual experience, which was around 16–17 years old for all groups.

#### 3.1.4 Sexual violence

Bisexual (64.9%) and heterosexual (54.5%) women were more likely to report having accepted sexual intercourse without truly wanting it compared to lesbians (34.9%). Bisexual women were significantly more likely to report regretting sexual encounters and experiences of sexual abuse, even though the number of lesbians sharing that experience is still quite high.

#### 3.1.5 Substance use and mental health

YSMW were significantly more likely to report current cannabis and tobacco use compared to heterosexual women. No significant differences were observed for alcohol misuse and illegal drugs. Trends were observed for mental health, with YSMW showing higher rates of mental health issues.

### 3.2 Multivariate analysis

Compared to heterosexual women, lesbians had significantly higher odds of reporting a later age at first gynecologist visit [RRR: 1.42 (1.22–1.65)] and engaging in three-way sexual encounters [RRR: 5.04 (1.58–16.02)], and lower odds of reporting the use of protection [RRR:0.01 (0.004–0.05)] or contraception [RRR:0.01 (0001–0.07)], and experiencing sexual intercourse with more than three partners at once [RRR:<.01 (<.01–0.00001)] (Table 2). Trends were observed indicating higher odds of currently smoking [RRR:2.98 (1.12–7.93)] and lower odds of having had fewer than four sexual partners in their lifetime.

**Table 2:** Results of the multivariate analysis.

Variables	Lesbians RRR (CI 99%)	Bisexual women RRR (CI 99%)	YSMW OR (CI 99%)
Sexual health			
Ever received STI diagnosis	0.74 (0.13; 4.05)	1.75 (1.09; 2.81) ^a^	1.36 [0.87–2.11]
HIV testing	0.68 (0.23–1.98)	0.90 (0.55–1.48)	
Mean age first gynecological consultation	1.42 (1.22; 1.65)*	1.00 (0.93; 1.08)	
Sexual behaviors			
Contraception/protection last intercourse			
Condom ± Contraceptive	0.01 (0.004; 0.05)*	0.19 (0.11; 0.33)*	0.12 (0.07–0.18)*
Contraceptive	0.01 (0.001; 0.07)*	0.22 (0.13; 0.40)*	0.13 (0.08–0.20)*
None	REF	REF	REF
Number of lifetime sex. partners			
0–3	0.21 (0.05; 0.81)^a^	0.67 (0.33; 1.37)	0.67 (0.39–1.15)
4–7	REF	REF	REF
8 or more	0.53 (0.07; 3.71)	1.07 (0.51; 2.23)	0.92 (0.50–1.68)
Number of lifetime occasional sex. partners			
0–3	2.65 (0.53; 13.30)	1.01 (0.50; 2.05)	1.20 (0.66–2.20)
4–7	REF	REF	REF
8 or more	5.29 (0.73; 38.11)	0.70 (0.38; 1.27)	0.80 (0.45–1.42)
Three-way	5.04 (1.58; 16.02)*	3.82 (2.12; 6.88)*	3.81 (2.27–6.42)*
More than 3 sex. partners at once	<0.01 (<0.01–0.0001)*	2.42 (1.07; 5.50)^a^	1.93 (0.87–4.30)
Sexual violence			
Sexual abuse	0.39 (0.12; 1.27)	3.07 (1.92; 4.93)*	2.21 (1.44–3.40)*
Regretted intercourse	1.21 (0.40; 3.67)	1.29 (0.75; 2.20)	1.26 (0.80–1.96)
Intercourse without wanting to	0.54 (0.19; 1.56)	1.06 (0.67; 1.67)	
Substance Use and Mental Health			
Cigarettes (yes)	2.98 (1.12; 7.93)^a^	1.51 (0.96; 2.37)	1.95 (1.31–2.90)*
Cannabis (yes)	2.34 (0.75; 7.27)	1.44 (0.81; 2.56)	1.44 (0.86–2.42)
Mental health (poor)			1.21 (0.77–1.90)

The multivariate analysis is controlled for the type of current relationship (one steady partner, one casual partner, several casual partners, no partner).

RRR, relative risk ratio; OR, odd ratio; CI, confidence interval; YSMW, young sexual minority women (grouping lesbians and bisexual women).

The significant results (*p* < .01) are marked with an *, and the trends (*p* < .05) are marked with a superscript^a^.

Compared to heterosexual women, bisexual women had significantly higher odds of engaging in three-way sexual encounters [RRR:3.82 (2.12–6.88)] and experiencing sexual abuse [RRR:3.07 (1.92–4.93)], and lower odds of reporting the use of protection (RRR:0.19 [0.11–0.33] or contraception [RRR:0.22 (0.13–0.40)]. Trends were observed for higher odds of reporting STI diagnosis [RRR:1.75 (1.09–2.81)] and engaging in sexual intercourse involving more than three sexual partners at once [RRR: 2.42 (1.07–5.50)].

### 3.3 Grouped analyses

To account for the small sample size of lesbian- and bisexual-identifying women, the same analyses were conducted on a combined group encompassing both subgroups. Overall, the results of these grouped analyses were similar to the results observed for the separate groups. However, analyzing the groups separately provided more detailed insights into the distinct health and behavior patterns of lesbians and bisexual women. For example, the separated analyses highlighted the fact that lesbians first visit a gynecologist at a later age (19.4) compared to both heterosexual and bisexual women, whereas the mean age was of 16.9 when they were grouped together. A similar observation can be made for the sexual abuse variable. While grouped results indicated that YSMW reported higher rates of sexual abuse compared to heterosexual women, the separate analyses highlighted that bisexual women reported significantly more sexual abuse, whereas lesbians reported rates similar to heterosexual women. Finally, the separate analysis, although this result was a trend, revealed that bisexual women reported more STI diagnoses than lesbians and heterosexual women.

## 4 Discussion

This study provides valuable insights into the sexual health, behaviors, and experiences of bisexual, lesbian, and heterosexual women, highlighting key differences and trends across these groups.

As shown in our results, YSMW are more likely not to use any kind of contraceptive or protective barrier. This result aligns with previous findings (11, 32). However, we must consider that the use of protective or contraceptive barriers might vary depending on the partner's sex or the nature of the relationship (steady or casual). One possible explanation for the lower rate of protection is that YSWM's sexual relations, especially between cisgender women, are often overlooked in protective promotion campaigns (20), which can spread the belief that WSW are less at risk of contracting a STI (6). Another factor to consider is the absence of the risk of unwanted pregnancy in a cisgender WSW relationship, which can reduce the use of protective contraception barriers. A systematic review on cling film and dental dam use during oral sex revealed challenges in their use such as misperceptions, lack of knowledge, and a preference for STI testing over such protective methods (33). These findings emphasize the need for comprehensive sexual health education that normalizes the use of protective barriers in all types of relationships. This approach would help ensure that such methods are not overlooked and instead become an integral part of the protective landscape in sexual health practices. Interestingly, the review also noted that younger and less sexually experienced women were more open and receptive to the possibility of using these methods in the future (33), highlighting a promising opportunity for targeted education and intervention efforts within this group. In this line, even though it is only a trend (*p* < .05) at the bivariate level, YSMW, particularly bisexual women, reported higher rates of having no source of sexual education. Additionally, bisexual women were more likely to report actively seeking information about sexuality. Beliefs and knowledge held by healthcare professionals also impact this lack of information, as put forth by a study led in France that highlighted the less frequent conversations regarding STI prevention with WSW than presumed heterosexual women (21). This suggests that YSMW would benefit from access to more comprehensive information on topics such as relationships between women or AFAB partners, sexual identity, and bisexual behaviors. This could be achieved through targeted prevention campaigns or more inclusive and informed sexual education in schools. As these women likely attended sex education classes in the late 2000s, it is possible that these programs were still too heteronormative, lacked adequate resources, or were constrained by limited time, resulting in insufficient inclusivity of diverse sexualities. Research that better focuses on SMW's needs in terms of sexual health and sexual practices is needed as well (34) which could bring a better understanding of their use of protective practices and help rethinking care approaches that are better suited to the needs of SMW.

We also found a trend showing that bisexual women reported a higher history of STI compared to heterosexual women and lesbians who reported the least. As shown in previous studies (9, 22), this may be attributed to bisexual women having a higher number of male partners than heterosexual women and less consistent use of protective barriers (11). Additionally, it is possible that women with a higher number of male sexual partners, regardless of sexual orientation, are more likely to undergo STI screening (1). While this specific information is not available in our dataset, a higher frequency of screening could potentially explain why YSMW report a higher rate of STI history. Based on our HIV testing variable, this appears to be a plausible explanation, as bisexual women reported the highest rates of testing.

Bisexual and heterosexual women first visit to a gynecologist happened at a younger age compared to lesbians, suggesting that their first consultations might be motivated by their first sexual encounters. A 2019 study conducted in the French-speaking part of Switzerland, focusing on YSMW aged 17–73, found that 28% of participants did not have a gynecologist (5). However, that study did not explore the frequency of visits or whether consultations occurred regularly - data that are also absent from the present study. It is possible that visiting a gynecologist at a younger age leads YSMW to consult with greater frequency. The opposite might also be true; YSMW often experience discrimination in medical settings, which discourage them from seeking care regularly (1, 35).

Bisexual women and lesbians were more likely to report having had threesomes, while a trend (*p* < .05) was observed for bisexual women regarding sexual encounters with more than three partners at once. It could be that, in experimenting with their sexuality, YSMW may be more curious about sexual encounters involving two or more partners of different genders. Regarding bisexuality, another possibility to consider is the oversexualization of self-identified bisexual women, which often leads their partners or interested parties, particularly straight men, to request a threesome (36). However, both hypotheses should be interpreted with caution, as bisexual individuals frequently face the stereotype of being more promiscuous than their heterosexual or lesbian counterparts (37). Furthermore, our data does not provide additional information about the context of these sexual encounters, such as consent, desire, or other relevant factors.

At the bivariate level, when considering YSMW as a combined group, they reported higher rates of victimization for both sexual abuse and regretted encounters compared to heterosexual women. However, when separating bisexual women and lesbians, it becomes evident that these elevated rates of sexual victimization are specifically driven by bisexual women, across all forms of sexual violence. At the multivariate level, only sexual abuse remained significant, but bisexual women showing a threefold higher risk compared to heterosexual women. A systematic review further highlights that bisexual women experience more sexual assault compared to heterosexual women (17), women exclusively attracted to women (16), and sexual minority men, placing them at the intersection of homophobia and sexism (38). Researchers have hypothesized that their oversexualization might contribute to their increased victimization (37, 39). However, further research is needed to fully explore how sexual orientation influences sexual victimization and the mechanisms underlying these disparities (39).

In accordance with the literature (10, 23), YSMW were more likely to smoke cigarettes when analyzed as a grouped category, but this association became only a trend when examined separately and was observed exclusively among lesbians. The difference in alcohol consumption was not significant which contradicts the findings by Blosnich and colleagues (10) and the National LGBT-Health Survey (35). A possible explanation for this discrepancy could be the age of the population in our study. As it is a sample of young adults, alcohol consumption might be normalized, for example in the context of partying or social drinking, an explanation also advanced in a study on bisexual and lesbian college students (3).

While sexual minorities generally present poorer mental health, bisexual women, in particular, have been shown to experience worse symptoms compared to both heterosexual women and lesbians (12). However, this pattern was not observed in our study, although at the bivariate level, both lesbians and bisexual women reported poorer mental health compared to heterosexual women, which aligns with some findings in literature as put forward by Ross and colleagues (40). Many sexual minority individuals may feel marginalized by the heterosexual and cisgender population; however bisexual individuals may also face marginalization within the LGBTQ community (12, 41). This lack of affirmed bisexual community has led some researchers to believe that the feelings of isolation could contribute to a higher risk of poor mental health (8, 10–12). As aptly put by Elia (42), “poor health outcomes have nothing to do inherently with bisexuality”, but is rather a consequence of the existence of bi-phobia, or bi-negativity. While both minority groups (gay/lesbian and bisexual) face challenges related to their sexuality, these challenges are distinct and unique to each population (12).

### 4.1 Strengths and limitations

The first strength of this study is its sample size. Even though the overall response rate was low (15.1%), the sample remains substantial for a study on sexual health among young adults. Moreover, the response rate is similar to that reported in other surveys on sexuality using similar methodologies (43). Few studies (9, 22, 23) focus on STI transmission and sexual risk behaviors among cisgender women, and more specifically non-heterosexual cisgender women. This research a more detailed examination of the most commonly reported risk behaviors among YSMW and identifies areas where prevention efforts are needed, while also highlighting avenues for future research.

However, some limitations should be acknowledged. Sexual health and behaviors are sensitive topics, and potential participants may have felt uncomfortable answering a web-based survey, even if it was secured. Moreover, participants were contacted exclusively by postal mail, which required them to access website and introduce a code to participate. This step may have reduced response rates compared to electronic invitations. The timing of the survey, conducted between June and September—Switzerland's summer holiday period—may have further decreased participation.

The present study lacks some data. For instance, details on the frequency of visits to the gynecologist, if there were any. We do not know if there is a difference in visits between heterosexual and YSMW in our sample, and if the frequency of those visits would be linked to a higher number of male sexual partners. Similarly, the data on the use of contraceptive and/or protective barriers does not indicate if the partner was a cisgender man or woman, an indication which influences the uses of protective methods.

An additional limitation of this study is the small sample size of YSMW, particularly lesbians, which limits the generalizability of findings for this subgroup. This highlights the need for more research focusing on lesbians to better understand their specific health and sexual behaviors. Moreover, it is possible that some individuals who identify as lesbians may still engage in sexual practices with partners of the opposite sex, which could influence the reported use of protective methods and risk behaviors. This underlines the importance of capturing nuanced information on sexual practices in future studies to provide a more comprehensive understanding of this population.

## 5 Conclusion

This study highlights key differences in the sexual health, behaviors, and experiences of young bisexual, lesbian, and heterosexual women. YSMW were less likely to use contraception or protective barriers during sexual encounters, emphasizing the need for inclusive sexual health education that normalizes protective methods in all relationships. Bisexual women were disproportionately vulnerable to sexual abuse, underscoring the importance of tailored prevention strategies addressing their specific risks and challenges. The small sample size of YSMW, especially lesbians, points to the need for further research into their unique health needs, while recognizing that sexual practices may not always align with sexual self-identity.

Excluding YSMW from prevention and sex education campaigns leaves a critical gap in addressing their needs. Future efforts must provide inclusive, comprehensive information on topics such as same-gender relationships, bisexual behaviors, and diverse sexual identities to reduce disparities in sexual and mental health outcomes.

## Data Availability

The data analyzed in this study is subject to the following licenses/restrictions: for this specific study, the dataset can be made available upon reasonable request to the corresponding author, subject to ethical considerations and data protection regulations. Requests to access these datasets should be directed to udd.data@unisante.ch or https://data.unisante.ch/index.php/catalog/31.
