# Supplementary material for: Sexual and mental health disparities among young sexual minority women compared to exclusively heterosexual women: a national study
Source: Front Glob Womens Health. 2026 Jan 20;6:1591604. doi: 10.3389/fgwh.2025.1591604 (PMC12864464; doi:10.3389/fgwh.2025.1591604)
Supplement: Supplementary file 1 [file Table1.docx]

|  | | | | | | |
| --- | --- | --- | --- | --- | --- | --- |
|  | | | | | | |
| Supplementary Material | | | | | | |
| Table 1. Results of the bivariate analyses | | | | | | |
|  | **Heterosexual women**  **(%) N = 2185** | **Lesbians**  **(%) N=28** | **Bisexual women**  **(%) N=103** | **p-value** | **YSMW**  **(%) N=131** | **p-value** |
| **Sociodemographic variables** | | | | | | |
| Age (mean ± s.e.) | 26.3 ±.02 | 26.4 ±.16 | 26.4 ±.08 | ns | 26.4±.07 | ns |
| Birthplace (Switzerland) | 88.4 | 87.4 | 82.3 | ns | 83.4 | ns |
| Family SES at age 15   - Above average - Average - Below average | 19.6  64.5  16.0 | 11.9  68.8  19.3 | 20.3  56.7  23.0 | ns | 18.6  59.2  22.2 | ns |
| Education level   - Apprenticeship - Secondary - Higher - Other | 19.4  19.9  56.3  4.3 | 28.9  24.8  40.5  5.8 | 26.0  24.4  43.9  5.7 | ns | 26.6  24.5  43.1  5.7 | <.05 |
| **Sexual health** | | | | | | |
| Ever received STI diagnosis | 13.4 | 5.8 | 26.4 | < .01 | 21.9 | <.01 |
| Chlamydia* | 16.9 | 0.0 | 28.9 | < .05 | 25.5 | Ns |
| Gonorrhea* | 0.9 | 11.0 | 0.0 | < .01 | 1.3 | Ns |
| Syphilis* | 0.1 | 0.0 | 0.0 | Ns | 0.0 | Ns |
| HPV* | 15.0 | 0.0 | 20.1 | Ns | 17.8 | Ns |
| Herpes* | 9.8 | 0.0 | 9.4 | Ns | 8.4 | Ns |
| Other* | 7.2 | 16.6 | 7.0 | ns | 8.1 | Ns |
| Ever tested for HIV | 48.6 | 38.5 | 62.2 | < .01 | 56.9 | Ns |
| HPV vaccine | 51.5 | 42.4 | 51.8 | ns | 50.1 | Ns |
| Gynaecologists visit at least once | 97.4 | 86.6 | 96.5 | < .01 | 94.4 | <.05 |
| Age at first gynaecologist visit (mean ± s.e.) | 16.8 ±.06 | 19.4 ±.55 | 16.3 ±.28 | < .01 | 16.9±.26 | Ns |
| **Sexual behaviors** | | | | | | |
| Age at first sexual experience (mean ± s.e.) | 16.5 ±.06 | 17.1 ±.47 | 15.9 ±.27 | ns | 16.1±.24 | ns |
| Contraception / protection last intercourse   - Condom +/- Contraceptive - Contraceptive - None | 51.2  39.9  8.9 | 9.3  3.2  87.5 | 37.5  36.8  25.7 | < .01 | 31.5  29.7  38.8 | <.01 |
| Number of lifetime sex. partners   - 1-3 - 4-7 - 8 or more | 41.1  26.5  32.4 | 34.1  24.8  31.0 | 17.9  25.5  56.6 | < .01 | 21.4  27.5  51.1 | < .01 |
| Number of occasional sex. partners   - 0-3 - 4-7 - 8 or more | 68.8  16.3  14.9 | 73.1  9.1  17.8 | 48.4  24.8  26.8 | < .01 | 53.7  21.4  24.8 | < .01 |
| Three-way | 7.6 | 19.6 | 33.7 | < .01 | 30.7 | < .01 |
| More than 3 sexual partners at once | 1.7 | 0.0 | 9.8 | < .01 | 7.7 | < .01 |
| Sexual intercourse under the influence of drugs or alcohol | 45.1 | 41.4 | 58.1 | < .05 | 54.5 | < .05 |
| Looked for sexual information | 23.2 | 15.3 | 32.3 | < .05 | 28.7 |  |
| The information was useful (n=545 had ever looked for sexual information)   - A lot - A little - Not at all - I don’t know | 31.0  57.7  9.1  2.1 | 22.9  77.1  0.0  0.0 | 32.4  54.8  10.2  2.6 | ns | 31.3  57.4  9.0  2.3 | ns |
| Main source of sex education during adolescence   - Parents - School - Friends - No one - Internet - Other | 31.3  16.4  37.0  2.4  5.3  7.5 | 31.9  14.9  26.4  0.0  18.4  8.4 | 29.1  14.1  39.2  6.7  5.0  5.8 | < .05 | 29.7  14.3  36.5  5.2  7.9  6.4 | ns |
| **Sexual violence** | | | | | | |
| Sexual abuse | 14.5 | 16.2 | 39.7 | < .01 | 34.6 | < .01 |
| Regretted intercourse | 51.4 | 48.1 | 74.5 | < .01 | 68.8 | < .01 |
| Intercourse without wanting to | 54.5 | 34.9 | 64.9 | < .01 | 58.4 | ns |
| **Substance use and mental health** | | | | | | |
| Current cigarettes smoker (yes) | 38.6 | 68.8 | 64.6 | < .01 | 65.5 | <.01 |
| Alcohol misuse 30 days (yes) | 26.4 | 25.3 | 30.3 | ns | 29.2 | ns |
| Cannabis use 30 days (yes) | 9.2 | 15.0 | 20.7 | < .01 | 19.5 | < .01 |
| Illegal drugs 30 days (yes) | 2.0 | 6.5 | 4.1 | ns | 4.6 | <.05 |
| Mental health (poor) | 16.7 | 26.5 | 24.6 | < .05 | 25.0 | <.01 |

* The percentages presented for each STI refer to individuals who reported having previously received a diagnosis: 285 heterosexual, 2 bisexual, and 26 bisexual or 28 YSMW.

ns: non-significant

s.e. : standard errors

YSMW: young sexual minority women (grouping lesbians and bisexual women)

| Table 2. Results of the multivariate analysis. | | |  |
| --- | --- | --- | --- |
|  |  |  |  |
| **Variables** | **Lesbians RRR**  **(CI 99%)** | **Bisexual women**  **RRR (CI 99%)** | **YSMW**  **OR (CI 99%)** |
| **Sexual health** | | |  |
| Ever received STI diagnosis | 0.74 (0.13 ; 4.05) | 1.75 (1.09 ; 2.81) ^a^ | 1.36 [0.87-2.11] |
| HIV testing | 0.68 [0.23-1.98] | 0.90 [0.55-1.48] |  |
| Mean age first gynecological consultation | 1.42 (1.22 ; 1.65)* | 1.00 (0.93 ; 1.08) |  |
| **Sexual behaviors** | | | |
| Contraception / protection last intercourse   - Condom +/- Contraceptive - Contraceptive - None | 0.01 (0.004 ; 0.05)*  0.01 (0.001 ; 0.07)*  REF | 0.19 (0.11 ; 0.33)*  0.22 (0.13 ; 0.40)*  REF | 0.12 [0.07-0.18]*  0.13 [0.08-0.20]*  REF |
| Number of lifetime sex. partners   - 0-3 - 4-7 - 8 or more | 0.21 (0.05 ; 0.81)^a^  REF  0.53 (0.07 ; 3.71) | 0.67 (0.33 ; 1.37)  REF  1.07 (0.51 ; 2.23) | 0.67 [0.39-1.15]  REF  0.92 [0.50-1.68] |
| Number of lifetime occasional sex. partners   - 0-3 - 4-7 - 8 or more | 2.65 (0.53 ; 13.30)  REF  5.29 (0.73 ; 38.11) | 1.01 (0.50 ; 2.05)  REF  0.70 (0.38 ; 1.27) | 1.20 [0.66-2.20]  REF  0.80 [0.45-1.42] |
| Three-way | 5.04 (1.58 ; 16.02)* | 3.82 (2.12 ; 6.88)* | 3.81 [2.27-6.42]* |
| More than 3 sex. partners at once | <0.01 [<0.01-0.0001]* | 2.42 (1.07 ; 5.50) ^a^ | 1.93 [0.87-4.30] |
| **Sexual violence** | | | |
| Sexual abuse | 0.39 (0.12 ; 1.27) | 3.07 (1.92 ; 4.93)* | 2.21 [1.44-3.40]* |
| Regretted intercourse | 1.21 (0.40 ; 3.67) | 1.29 (0.75 ; 2.20) | 1.26 [0.80-1.96] |
| Intercourse without wanting to | 0.54 (0.19 ; 1.56) | 1.06 (0.67 ; 1.67) |  |
| **Substance Use and Mental Health** | | | |
| Cigarettes (yes) | 2.98 (1.12 ; 7.93) ^a^ | 1.51 (0.96 ; 2.37) | 1.95 [1.31-2.90]* |
| Cannabis (yes) | 2.34 (0.75 ; 7.27) | 1.44 (0.81 ; 2.56) | 1.44 [0.86-2.42] |
| Mental health (poor) |  |  | 1.21 [0.77-1.90] |

The significant results (p<.01) are marked with an *, and the trends (p<.05) are marked with a superscript ^a^.

The multivariate analysis is controlled for the type of current relationship (one steady partner, one casual partner, several casual partners, no partner).

RRR: relative risk ratio

OR: odd ratio

CI: confidence interval

YSMW: young sexual minority women (grouping lesbians and bisexual women)
